# Reduced spontaneous itch in mouse models of cholestasis

**DOI:** 10.1038/s41598-021-85660-1

**Published:** 2021-03-17

**Authors:** Jacqueline Langedijk, Ruth Bolier, Dagmar Tolenaars, Lysbeth ten Bloemendaal, Suzanne Duijst, Dirk de Waart, Ulrich Beuers, Piter Bosma, Ronald Oude Elferink

**Affiliations:** Amsterdam UMC, Tytgat Institute for Liver and Intestinal Research, Amsterdam Gastroenterology Endocrinology Metabolism Research Institute, University of Amsterdam, Meibergdreef 69-71, 1105 BK Amsterdam, The Netherlands

**Keywords:** Biochemistry, Biological techniques, Physiology, Biomarkers, Diseases, Gastroenterology, Molecular medicine, Signs and symptoms

## Abstract

Pruritus is one of the most distressing symptoms in cholestatic patients. Plasma autotaxin (ATX) activity correlates with the severity of pruritus in cholestatic patients, but the pathophysiology is unclear. To study pruritus in mice, we measured scratch activity in cholestatic *Atp8b1* mutant mice, a model for Progressive Familial Intrahepatic Cholestasis type 1, and wild type mice (WT) with alpha-naphthylisothiocyanate (ANIT)-induced cholestasis. To induce cholestasis, *Atp8b1* mutant mice received a diet containing 0.1% cholic acid (CA) and WT mice were treated with ANIT. In these mice ATX was also overexpressed by transduction with AAV-ATX. Scratch activity was measured using an unbiased, electronic assay. Marked cholestasis was accomplished in both *Atp8b1* mutant mice on a CA-supplemented diet and in ANIT-treatment in WT mice, but scratch activity was decreased rather than increased while plasma ATX activity was increased. Plasma ATX activity was further increased up to fivefold with AAV-ATX, but this did not induce scratch activity. In contrast to several reports two cholestatic mouse models did not display increased scratch activity as a measure of itch perception. Increasing plasma ATX activity by overexpression also did not lead to increased scratch activity in mice. This questions whether mice are suitable to study cholestatic itch.

## Introduction

Cholestasis is defined by impaired bile flow due to intra- or extrahepatic causes. One of the most distressing symptoms associated with cholestasis is pruritus (itch). Chronic pruritus leads to loss of concentration, sleep problems and sometimes even suicidal ideations. Existing treatment options have limited and variable efficacy. The pathophysiology of cholestatic pruritus remains unclear. We postulated that cholestatic pruritogens are biliary compounds that accumulate in plasma and are transferred to the skin to cause pruritus^1^. To further investigate this mechanism and test possible interventions, we aimed to measure itch perception in mouse models of cholestatic pruritus, using scratch activity as an outcome measure.

One of the diseases leading to cholestasis in humans is progressive familial intrahepatic cholestasis type I (PFIC I), which is caused by a mutation in the *ATP8B1* gene. This leads to a mutant FIC1 protein that is rapidly broken down. One of the mouse models we used is the *Atp8b1* mutant mouse, the animal model for progressive familial intrahepatic cholestasis type 1. These animals bear the homozygous G308V mutation in *Atp8b1*, orthologous to that present in patients with PFIC1^2^. ATP8B1 is a flippase responsible for inward phospholipid translocation across membranes and is essential for normal function of canalicular transporters^3^. Defective inward phospholipid translocation in the hepatocyte apical membrane impairs the bile salt export pump (BSEP), leading to intrahepatic cholestasis^4^. PFIC1 patients often suffer from severe pruritus. *Atp8b1*^*G308V/G308V*^ mice have very mild cholestasis, but upon treatment with a diet supplemented with cholic acid, they develop severe cholestasis, as witnessed by elevated plasma alkaline phosphatase (ALP), bile salt and bilirubin levels. Itch perception has never been studied in these mice.

As a second model for cholestasis, we used treatment of wild type mice with alpha-naphthylisothiocyanate (ANIT), which induces biliary cell injury and cholangitis^5,6^. Whether ANIT-induced cholestasis induces pruritus remains unclear, since Cipriani et al. found no increase of scratching behavior^7^, whereas Meixiong et al. claimed enhanced scratching activity^8^.

The plasma enzyme autotaxin (ATX) is a secreted lysophospholipase D that converts lysophosphatidyl-choline (LPC) into lysophosphatidic acid (LPA). In our previous studies, we have shown that plasma ATX protein concentration and activity are increased in cholestatic patients and that there is a striking correlation with itch intensity^9,10^. To study the relation between ATX and cholestatic pruritus, we measured ATX activity in the plasma of cholestatic mice as well as their scratch activity.

The overall aim of our study was to assess scratch activity in cholestatic mice, and to ascertain the potential role of ATX in induction of itch perception. A series of publications from different groups has reported scratch behavior in mouse models of cholestasis (mainly induced by bile duct ligation or treatment with ANIT)^8,11–13^. However, these publications measured scratch activity using short term camera registration and subjective assessment of scratch movements. We have developed an automated objective registration over periods of many hours during the night in order to monitor mice in their active phase and to avoid disturbance of their behavior^9^. During the present study, we discovered a decreased rather than increased scratch activity in *Atp8b1* mutant mice as well as in ANIT-treated WT mice during cholestasis. Serum ATX activity levels were increased in both mouse models, but this apparently did not cause itch. Further increase in circulating ATX levels by viral transduction of ATX also did not increase scratch activity in mice.

## Materials and methods

### Human subjects

Human plasma samples were collected from healthy volunteers, from cholestatic patients without pruritus, cholestatic patients with pruritus (including intrahepatic cholestasis of pregnancy (ICP)), and pregnant healthy volunteers after informed consent as reported previously^10,14^ in accordance with the Declaration of Helsinki. Patient sample collection was performed with approval of the medical ethical committee of the Academic Medical Center (METC AMC #NL21233.018.07). Informed consent was obtained from all patients.

### Experimental animals

Experiments were performed with C57Bl/6 wild type mice (Janvier) and *Atp8b1*^*G308V/G308V*^ mice^2^ (hereafter referred to as *Atp8b1* mutant).

The *Atp8b1* mutant mouse was used as a model of intrahepatic cholestasis. The mice received semi-synthetic reference diet supplemented with 0.1% cholic acid to intensify cholestasis. Control mice only received semi-synthetic reference diet. ATX expression was induced by tail vein injection of ± 1.4 × 10^14^ genomic copies (gc)/kg AAV8-CMV-ATX or ± 2.0 × 10^13^ gc/kg AAV8-HLP-ATX in male mice; the latter have higher transduction efficiency^15^.

Mice were housed conventionally with ad libitum water and food, consisting of regular chow (Teklad 2916, Envigo, isoflavone content: below 20 mg/kg), phytoestrogen-rich diet (Teklad 7912, Envigo, isoflavone content: 300–600 mg/kg) or semi-synthetic reference diet (20% casein, Arie Blok 4068.02) as indicated for different experiments, either or not supplemented with 0.1% cholic acid (CA, Sigma-Aldrich C1254). Pregnancy term was estimated by daily check of vaginal sperm plugs and, if in doubt, ultrasound.

All animal experiments were approved by the institutional committee for animal experiments (DEC licences ALC11 and ALC291) and licensed by the Dutch Central Committee for animal experiments (CCD licence AVD1180020172869). All methods were carried out in accordance with relevant guidelines and regulations. The study was also carried out in compliance with the ARRIVE guidelines (http://www.nc3rs.org.uk/page.asp?id=1357).

### Scratch activity assay

Scratch activity and total movements of the animals was measured using an in-house developed system adapted from^16,17^. Teflon coated 5 × 2 mm magnets (VWR European) were implanted subcutaneously under general anesthesia in both hind paws (below the knee) a week before the experiment. Accompanied by a littermate of the same gender without magnets, mice were placed in their normal cages and these were placed in specially fitted magnetic coils. An oscillograph attached to a computer registered the electric currents induced by movements of the implanted magnets. Long-term scratch activity and total movements were assessed at night (7 pm-7am) for the duration as described with each experiment.

Custom-made software was used to quantify paw movements in a given period of time according to adjustable settings for the following characteristics: frequency (in Hz), amplitude (outreach of the paw, mV), amount of consecutive beats (strikes of the paw) per scratch bout, and a maximal variation coefficient (CV_max_, %) of the amplitude of the beats within one bout. A scratch bout was defined by an event with a frequency between 10 and 20 Hz, an amplitude ≥ 300 mV, ≥ 4 beats per bout, and a CV_max_ of 35%. To validate our method, we assessed paw movement characteristics upon intradermal injection of the mast cell degranulator compound 48/80. This caused acute scratching for about 30 min (suppl. Figure 1a). We validated our method by simultaneous measurement with the magnetic coil system and video recording (suppl. Figure 1b). These experiments showed that the magnetic coil system has a sensitivity of 50% and a specificity of 94% for registration of scratch movements compared to video recording. In all other, chronic experiments, scratch activity was expressed as the average total duration (in seconds) of scratch movements per 12 h measurement. Total movements were defined by an event with a frequency of 0.1–3 Hz, amplitude ≥ 30 mV, ≥ 0 beats per bout, and a CV_max_ of 100%. Total movements were expressed as the average total duration (in seconds) of total movements per 12 h measurement.

### Plasma parameters

Blood was sampled by cheek vene puncture, collected in a heparin microvette tube (Sarstedt) and centrifuged at 2000xg for 5 min to isolate plasma. Cholestasis parameters were measured by the laboratory clinical chemistry department of the Amsterdam UMC according to standard procedures.

### Reagents

Compound 48/80 (Sigma) was dissolved in 0.9% NaCl to a concentration of 140 mg/mL. Mice received a single intradermal injection of 50µL 0.9% NaCl or compound 48/80 solution. ANIT (α-naphthylisothiocyanate; Sigma) was dissolved in corn oil to a concentration of 12.5 mg/mL. Mice received oral gavage of 100 µL (50 mg/kg) per day for five consecutive days. The ATX-inhibitor PAT-048^18^ was kindly donated by PharmAkea Therapeutics. It was dosed once daily by oral gavage (20 mg/kg/day) in a vehicle of 0.5% hydroxypropyl methylcellulose (Sigma-Aldrich 64,620) (3.3 mg/mL). Mice were injected intraperitoneally with 100 µL (40 mg/kg) per day for five consecutive days.

### Generation of AAV8-CMV-ATX and AAV8-HLP-ATX

Murine Atx cDNA (2588 bp), kindly provided by Dr. Boutin^19^, was inserted into pTRCGW behind the CMV promoter^20,21^ using XhoI and ApaI sites. In AAV8-HLP-ATX, the CMV promoter was replaced by a hybrid hepatocyte-specific promoter (HLP) using BsgrGI and XbaI sites^22^. AAV8 virus was produced in HEK293T cells using helper plasmid pDP8 (PlasmidFactory PF478) and purified using an iodixanol gradient^20,23^. Virus was injected via the tail vein in a dosis of ± 1.4 × 10^14^ gc/kg for AAV8-CMV-ATX and ± 2.0 × 10^13^ gc/kg for AAV8-HLP-ATX.

### Autotaxin activity assay

Plasma was incubated at 37 °C, with 1.25 mmol/L lysophosphatidylcholine 14:0 (LPC) (Avanti 855575C-1G) in 100 mmol/L Tris buffer (pH 9.0, BioRad 161-719) containing 500 mmol/L NaCl (Merck 6404), 5 mmol/L MgCl_2_ (Merck 5833) and 0.05% Triton X-100 (BioRad 161-0407)(plasma end dilution 1:40). After 60 min, the amount of choline generated by ATX was quantified using 2 U/mL choline-oxidase (Sigma-Aldrich C5896), 2500 U/mL peroxidase (Roche 1,010,809,000) and 2 mmol/L homovanillic acid (HVA, Sigma-Aldrich H-1252) in a 50 mmol/L 3-(N-morpholino)propanesulfonic acid (MOPS, VWR 443832 T) buffer (pH 8.0), containing 20 mmol/L CaCl_2_ (Merck 2389) and 0.1% Triton X-100 (BioRad 161-0407). ATX activity was determined by measuring fluorescence in a CLARIOstar analyser (BMG Labtech GmbH; excitation 320 nm, emission 405 nm).

### Autotaxin immunohistochemistry (IHC)

ATX expression in hepatocytes upon AAV transduction was determined in paraffin embedded mouse liver sections using a rat-anti-ATX monoclonal antibody (clone 4F1, kindly provided by J. Aoki) as previously described^24^. To detect 4F1, slides were first incubated with rabbit F(ab’)_2_ anti-rat IgG (H + L) (Southern Biotech 6130-01) and subsequently with a BrightVision poly-AP labelled anti-rabbit IgG (H + L) (Immunologic DPVR110AP).

### Statistical analyses

Assessment of long-term scratch activity was performed in groups of 6–10 mice. Differences between two groups under the same condition were tested by unpaired t-tests, differences within the same group under different consecutive conditions by paired t-tests, using GraphPad prism (version 8.0.2).

### Ethics approval

All animal experiments were approved by the institutional committee for animal experiments (DEC licences ALC11 and ALC291) and licensed by the Dutch Central Committee for animal experiments (CCD licence AVD1180020172869)). All methods were carried out in accordance with relevant guidelines and regulations. The study was carried out in compliance with the ARRIVE guidelines.

### Informed consent

Patient sample collection was performed with approval of the medical ethical committee of the Academic Medical Center (METC AMC #NL21233.018.07). Informed consent was obtained from all patients.

## Results

### Cholestasis in Atp8b1 mutant mice can be induced with 0.1% cholic acid diet

Intrahepatic cholestasis can be induced in *Atp8b1* mutant mice by feeding them a 0.1% CA supplemented semi-synthetic diet^2,24^. In this experiment, seven female *Atp8b1* mutant mice and seven wild type (WT) mice were treated for 14 days with 0.1% CA. Before treatment, they received a semi-synthetic reference diet without CA for at least four consecutive nights. In order to mimic cholestasis of pregnancy, WT and *Atp8b1* mutant mice were also studied during pregnancy (n = 6 and 7 females, respectively) with the latter group fed a 0.1% CA diet from day 12 of the pregnancy onwards. Increased concentrations of plasma alkaline phosphatase, total bile salts and bilirubin confirmed the presence of cholestasis in *Atp8b1* mutant mice (*p* < 0.01) (Table 1).

**Table 1:** Plasma parameters of non-pregnant and pregnant WT and *Atp8b1* mutant mice on the indicated diet.

	Wild type			Atp8b1 mutant		
Pregnancy	−	−	+	−	−	+
0.1% CA diet (days)	−	14	−	−	14	6
# of mice	13	7	6	14	7	7
ALP (U/L)	133.8 ± 39.8	86.2 ± 6.9	78.4 ± 13.4	207.9 ± 68.3	678.1 ± 166.5^a^	177.8 ± 100.3
Bile salts (μmol/L)	9.5 ± 5.3	5.9 ± 1.0	10.2 ± 3.9	79.3 ± 77.7	556.9 ± 318.6^a^	263.9 ± 216.7
Bilirubin (μmol/L)	ND	ND	ND	7.6 ± 11.2	88.6 ± 42.2^a^	21.4 ± 25.6

Diet was administered during the indicated period before measurement Numbers reflect mean ± SD.

*ND* not detectable.

Statistics: ^a^indicates significant (*p* < 0.01) increase compared to same genotype before introduction of the 0.1% CA diet (paired t-tests).

### ATX activity is increased in cholestatic Atp8b1 mutant mice, but scratch activity is decreased

**Figure 1:**
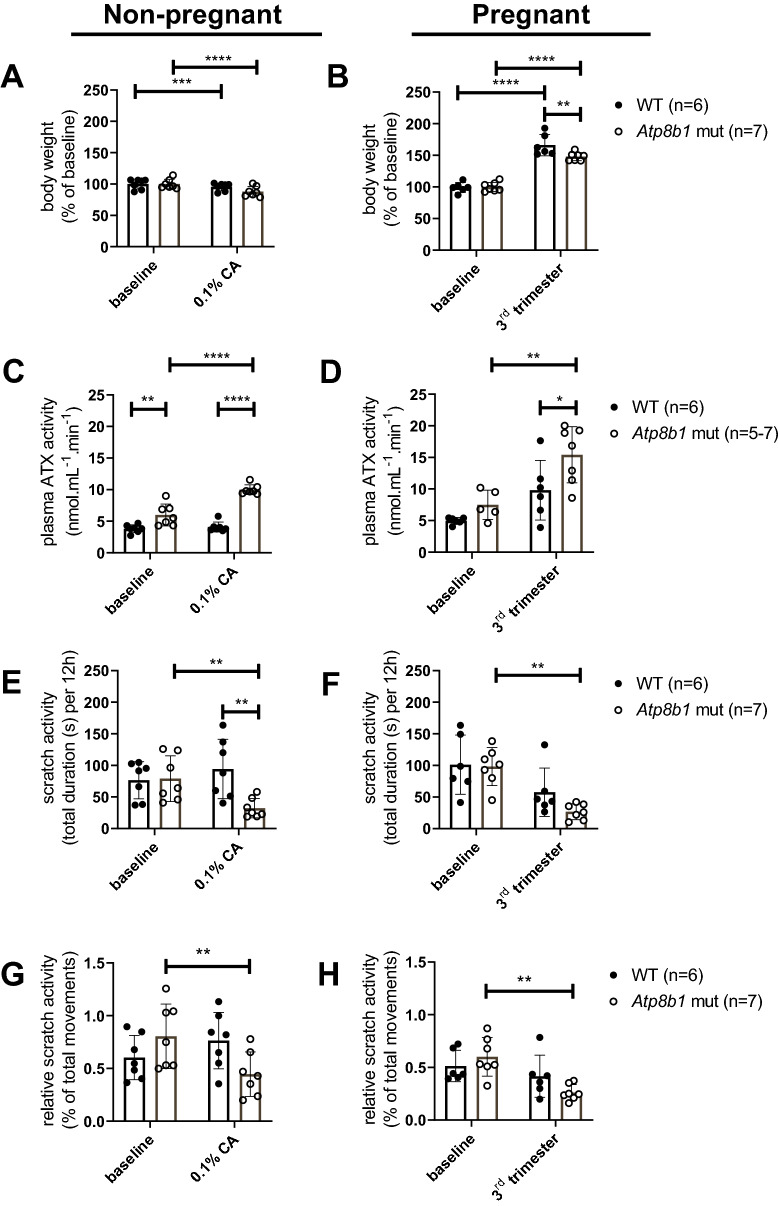
Body weight, plasma ATX activity and scratch activity in mouse models of cholestasis and cholestatic pregnancy. (**A,C,E,G**) Wild type mice (n = 7 females) and *Atp8b1* mutant mice (n = 7 females) fed with semi-synthetic reference diet for four days and subsequently with a 0.1% CA supplemented diet for 14 days. (**B,D,F,H**) Wild type mice (n = 6 females) on a semi-synthetic reference diet and *Atp8b1* mutant mice (n = 7 females) on a semi-synthetic reference diet with supplementation of 0.1% CA from day 12 of pregnancy, were observed before and during pregnancy. Body weight (**A,B**), plasma ATX activity (**C,D**) and mean scratch activity during 4 consecutive nights (**E,F**) were measured. To correct for a reduction in total movements during cholestasis and pregnancy, scratch activity is also displayed as a percentage of total time that the animals were moving (**G,H**). For total movements see Suppl. Figure 1. Plasma ATX activity data during pregnancy (**D**) is adjusted from^24^. Bars depict mean ± SD. Statistics: two-way ANOVA followed by Sidak’s post hoc test; within genotype all is shown, between genotypes only shown when significant; ns: not significant, *p-value < 0.05, **p-value < 0.01, ***p-value < 0.001, ****p-value < 0.0001.

*Atp8b1* mutant mice on a 0.1% CA diet reach their humane endpoint, weight loss > 15%, due to bile salt accumulation and hepatic injury^2^, after 3 weeks (suppl. Figure 2). Animal welfare regulations do not allow longer treatments. Figure 1A confirms that during 0.1% CA feeding, both W2T and *Atp8b1* mutant mice show a small but significant weight loss. During pregnancy, both genotypes gained weight, but *Atp8b1* mutant mice on 0.1% CA gained significant less weight than WT animals on reference diet (Fig. 1B).

By comparing WT and *Atp8b1* mutant mice on semi-synthetic reference diet with and without 0.1% CA, we noticed that plasma ATX activity was induced by feeding a 0.1% CA diet. In *Atp8b1* mutant mice on 0.1% CA for 2 weeks, plasma ATX activity rose from 6.0 ± 1.7 to 10.0 ± 0.8 nmol·mL^−1^·min^−1^ (167%, *p* < 0.0001), whereas the diet did not significantly induce plasma ATX in WT (108%, *p* = 0.87) (Fig. 1C). Total movements were already different between the groups at baseline and decreased significantly stronger in *Apt8b1* mutant mice upon 0.1% CA diet (77%) compared to WT mice (93%) (suppl. Figure 3A).

The induction of plasma ATX activity during pregnancy was comparable in WT mice on reference diet (197% from 5.0 ± 0.5 to 9.8 ± 4.7 nmol·mL^−1^·min^−1^, *p* = 0.06) and in *Atp8b1* mutant mice on 0.1% CA diet (206% from 7.5 ± 2.3 to 15.4 ± 4.5 nmol·mL^−1^·min^−1^, *p* = 0.002) (Fig. 1D).

Scratch activity was not increased in any of these models. In fact, *Atp8b1* mutant mice scratched less during cholestasis (41% of baseline, *p* = 0.005) and cholestatic pregnancy (27% of baseline, *p* = 0.001) (Fig. 1E,F). Total movements during pregnancy were decreased in both WT mice (69%) and *Atp8b1* mutant mice (60%) compared to the period before pregnancy (suppl. Figure 3A,B), indicating that pregnancy lowers mobility. In order verify whether the reduction in scratch activity is related to the reduction in total movements we also calculated scratch activity as a percentage of total movements (Fig. 1 G,H); this demonstrated that also the relative scratch activity was reduced in cholestatic Atp8b1 mutant mice. To further exclude that disturbed behavioral activity is the basis for a reduction in scratch activity, we intradermally injected the pruritogen compound 48/80 in both wild type and cholestatic Atp8b1 mutant mice (suppl. Figure 3C). Scratch activity was as strongly induced in the cholestatic mice as in the wild type mice, indicating that the reduced baseline scratch activity is not caused by impaired behavioral activity.

### Induction of cholestasis with ANIT treatment leads to reduced scratch activity in WT mice

To verify that the reduction of cholestasis-induced itch is not a specific result of the *Atp8b1* mutant mouse model, we also tested a second cholestatic model. C57Bl/6 mice (WT) treated with the cholestasis-inducing toxicant ANIT, were measured for their scratch activity and total movements. In a pilot study, WT mice were treated with 25 mg/kg/day for five consecutive days. In contrast to earlier reports^7,8,25^, this did not cause cholestasis and had no effect on scratch activity (not shown). Therefore, we increased the ANIT concentration to 50 mg/kg/day. First, baseline scratch activity was measured in all mice for five days (phase 1), then vehicle corn oil; 100 µL was orally administered per gavage (five days; phase 2) and subsequently six mice per group were treated with either vehicle or ANIT 50 mg/kg/day dissolved in corn oil given per gavage (five days; phase 3). This treatment 50 mg/kg/day ANIT for five days did lead to a significant increase in plasma ATX activity, bilirubin, aspartate aminotransferase (AST), alanine aminotransferase (ALT) and alkaline phosphatase (ALP) compared to vehicle treatment in phase 3 (suppl. Figure 4B,G).

**Figure 2:**
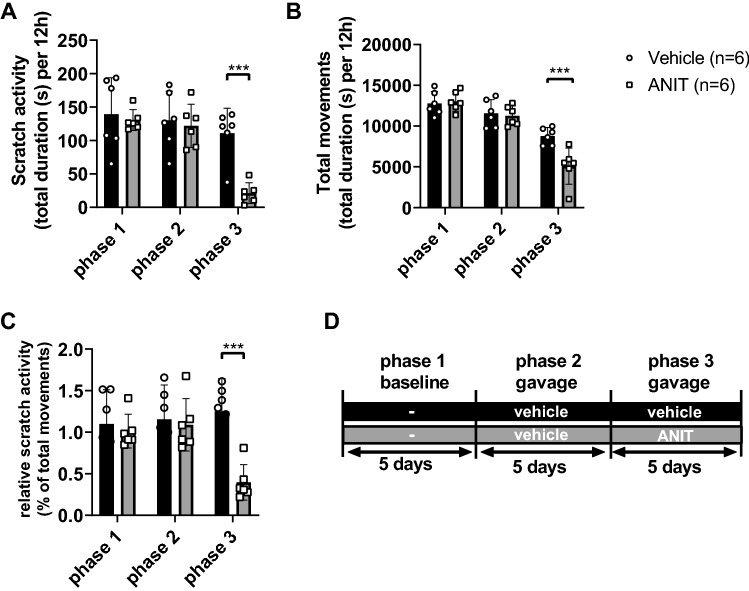
Scratch activity (**A**), total movements (**B**) and relative scratch activity as percentage of total movements (**C**) in WT mice during baseline (phase 1), gavage with vehicle (phase 2) and after treatment with vehicle (n = 6) or ANIT (n = 6) (phase 3). Panel (**D**) shows the experimental setup. Each bar represents the mean of six mice over five days ± SD. Statistics: two-way ANOVA followed by Sidak’s post hoc test. ***p-value < 0.001.

Figure 2 shows a strong decrease in scratch activity upon treatment with ANIT compared to treatment with vehicle in phase 3 (Fig. 2A). There was a concomitant but less strong decrease in total movements of the animals (Fig. 2B). This suggests a decrease in the wellbeing of the mice although body weight was not significantly reduced (suppl. Figure 4A). Taking the reduction of total movements in account by calculation of the relative scratch activity (as a percentage of total movements) showed that there still was a strong reduction in scratch behavior (Fig. 2C).

### Chow with phytoestrogens does not affect scratch activity in mice

**Figure 3:**
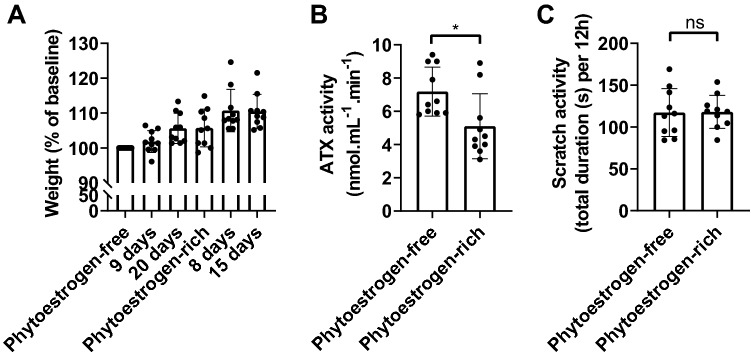
(**A**) Weights (as percentage of baseline), (**B**) ATX activity (after three weeks of phytoestrogen-free and three weeks of phytoestrogen-rich diet), (**C**) scratch activity (mean of four measurements during three weeks) in *Atp8b1* mutant mice (n = 10) on phytoestrogen-free and phytoestrogen-rich diet. Bars depict mean ± SD. Statistics: Paired t-test, ns: not significant; *p-value < 0.05.

One of the human cholestatic diseases that often presents with chronic itch is intrahepatic cholestasis of pregnancy (ICP) where pruritus can be extreme in the third trimester but rapidly resolves after delivery^26^. Higher circulating estrogen and progesterone levels compared to normal pregnancy values are associated with an increased frequency of ICP^27^. In some patients with a history of ICP, consumption of the combined oral contraceptive pill led to the development of pruritus^28^. In ICP patients treated with UDCA, urinary excretion of steroid hormone metabolites diminished, simultaneous to a decrease in pruritus^29^. Since estrogens induce ATX expression in rats^30^ and in man^14^, estrogen or estrogen metabolites could be involved in ATX induction resulting in cholestatic itch. To test whether scratch activity in mice would be influenced by estrogen, we fed ten mice first with regular phytoestrogen-free diet for three weeks and then with phytoestrogen-rich diet for three weeks (Teklad 7912). Teklad 7912 is based on soybean meal, containing 300 to 600 mg/kg isoflavone concentrations. Isoflavones belong to the group of phytoestrogens. In comparison, regular diet (Teklad 2916) does not contain soybean meal, thereby bringing the isoflavone concentrations below 20 mg/kg. Feeding *Atp8b1* mutant mice a regular phytoestrogen-free diet resulted in a minor cholestatic phenotype (Table 1). Figure 3 shows that diet containing high dose of phytoestrogen did not affect weight gain nor scratch activity while plasma ATX activity was reduced.

### Transduction of mice with AAV8-CMV-ATX and AAV8-HLP-ATX increases plasma ATX activity, but decreases scratch activity

**Figure 4:**
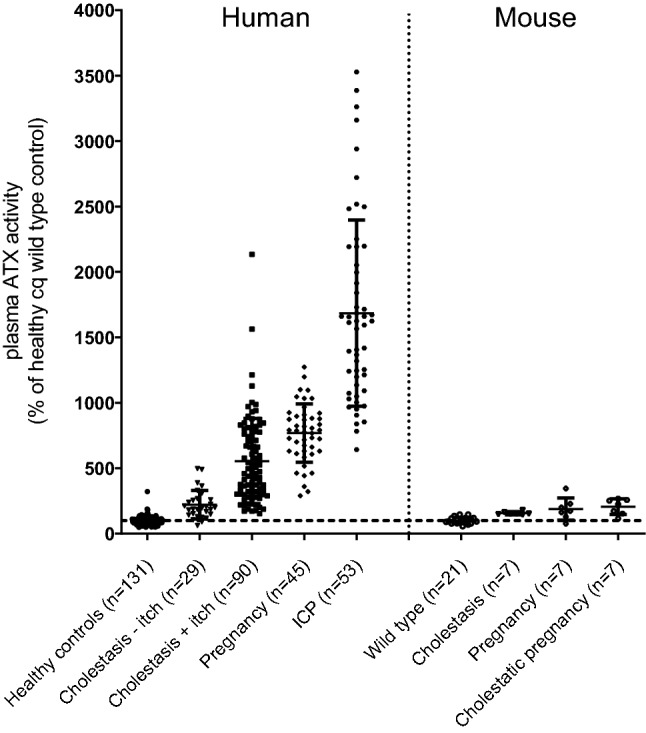
Plasma ATX activity relative to healthy control in man (left part of the figure) or wild type control and cholestatic Atp8b1 mutant mice (right part of the figure); human data adapted from^10,14^, mouse data adapted from Fig. 1).

Plasma ATX activity in cholestatic and pregnant *Atp8b1* mutant mice is increased, but compared to human patient groups, the values in mice are still very low (Fig. 4) (absolute values of control groups are comparable). The increase in cholestatic and pregnant mice is maximally 2.1-fold, whereas the increase in cholestatic and pregnant cholestatic humans is 5.5 and 16.8-fold. We hypothesized that the induction of plasma ATX activity in mice could be too small to cause itch, explaining the lack of increased scratch activity in mice.

**Figure 5:**
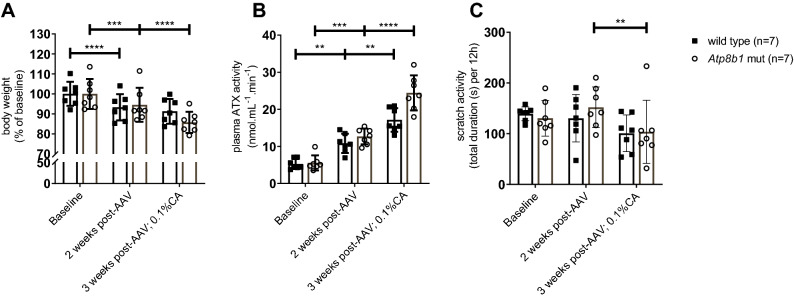
Inducing ATX activity by AAV8-CMV-ATX transduction does not increase scratch activity. Body weight (**A**), plasma ATX activity (**B**) and scratch activity (**C**) were measured during baseline, 2 weeks after AAV8-CMV-ATX injection and 3 weeks after AAV8-CMV-ATX injection combined with 0.1% CA diet for one week in both WT and *Atp8b1* mutant mice (n = 7). Bars depict mean ± SD. Statistics: paired t-test within groups, *indicates *p*-value < 0.05; **indicates *p*-value < 0.01.

To investigate this hypothesis an Adeno Associated Viral vector (AAV8)^31^ encoding mouse ATX, AAV8-CMV-ATX was used to establish higher plasma ATX values in (*Atp8b1* mutant) mice. Two weeks after the administration of ± 1.4 × 10^14^ gc/kg of AAV8-CMV-ATX, the mice received a diet containing 0.1% CA. Transduction of AAV-CMV-ATX resulted in a slight decrease in body weight after two weeks in WT and *Atp8b1* mutant mice (Fig. 5A). Cholic acid feeding further reduced body weight in *Atp8b1* mutant mice, to 85.5% of weight at the time of injection, but not in WT mice.

Liver transduction by AAV8-CMV-ATX increased plasma ATX activity in both WT and *Atp8b1* mutant mice, that was further increased upon cholic acid feeding (Fig. 5B). This 3.3 to 4.4-fold increase in ATX activity did not induce long-term scratch activity but in contrast a 33% decrease was observed (Fig. 5C).

**Figure 6:**
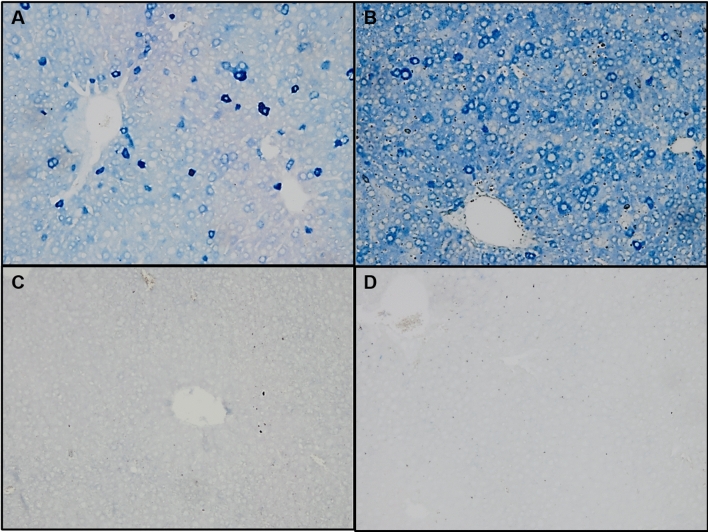
ATX immunohistochemistry of mouse livers after transduction with AAV8-CMV-ATX (**A**) and AAV8-HLP-ATX (**B**) constructs. (**C**) ATX is undetectable in non-transduced mouse liver. (**D**) Staining control using liver sample of the same mouse as in (**B**), leaving out the primary anti-ATX antibody. 10 × magnification for all pictures.

Although ATX plasma levels were increased in these mice, the levels are still relatively low compared to those seen in patients suffering from cholestasis. To further increase plasma ATX activity, we transduced WT mice (n = 13, chow diet) with an AAV8 construct containing a liver-specific promoter (HLP) that, compared to the CMV promoter, yields a higher expression in hepatocytes, the cells targeted by AAV8^22^. Figure 6A,B shows that indeed hepatic ATX protein expression after transduction with ± 2.0 × 10^13^ gc/kg of AAV8-HLP-ATX was more prominent than with AAV8-CMV-ATX. Specificity for staining of ATX was demonstrated by the absence of staining in non-transduced mice (Fig. 6C) and the absence of staining when the primary antibody was left out (Fig. 6D).

**Figure 7:**
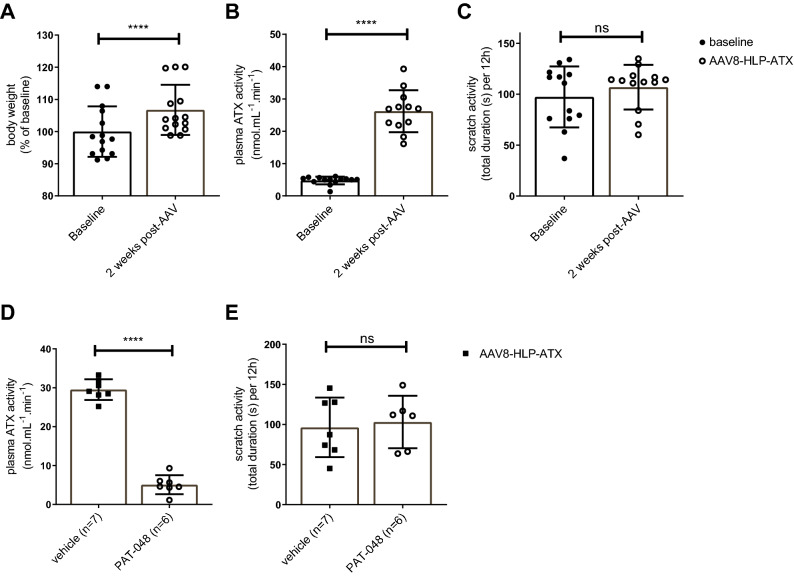
The effect of high efficiency hepatic AAV8-HLP-ATX transduction in WT mice. Body weight (**A**) and plasma ATX activity (**B**) were measured at baseline and 2 weeks after AAV-HLP-ATX injection in WT mice (n = 13). Scratch activity (**C**) was measured during four consecutive nights at baseline and 2 weeks after AAV-HLP-ATX injection in WT mice (n = 13). Plasma ATX activity (**D**) was measured 28 days after AAV and 24 h after the 4th dose of the ATX-inhibitor PAT-048 (oral gavage 20 mg/kg/day, n = 6) and vehicle (n = 7). Scratch activity (**E**) was measured as the mean of day 24–27 post-AAV injection, during oral gavage of PAT-048 (20 mg/kg/day, n = 6) and vehicle (n = 7). Bars depict mean ± SD. Statistics: paired t-test within groups (**A,B,C**), unpaired t-test between groups (**D,E**), ns: not significant, ****indicates *p*-value < 0.0001.

Hepatic ATX overexpression resulted in a small increase in body weight (6%, *p* < 0.0001, paired t-test)) (Fig. 7A) indicating that it did not adversely affect animal health. In line with the higher hepatic ATX protein expression, 3 weeks after transduction plasma ATX activity was increased to a higher level than obtained with AAV8-CMV-ATX: 5.5-fold (Fig. 7B; from 4.8 ± 1.2 to 26.2 ± 6.5 nmol·mL^−1^·min^−1^ (*p* < 0.0001)). This increase in plasma ATX activity, 550% of wild type control, is comparable to the increase observed in cholestatic patients with itch (Fig. 4). Again, however, these high plasma levels did not affect scratch activity significantly (*p* = 0.31, Fig. 7C). To check if the decrease (or lack of increase) in scratch activity was due to the higher plasma ATX activity, six mice that were treated with AAV8-HLP-ATX received the ATX-inhibitor PAT-048^18^, and seven were given a vehicle control. Oral administration of PAT-048 (20 mg/kg) suppressed plasma ATX activity by 82.8% (Fig. 7D, p < 0.001), but did not affect scratch activity (Fig. 7E).

## Discussion

Cholestatic itch is one of the most burdensome symptoms for cholestatic patients but the pathophysiology is still unclear. We have reported that itch intensity correlates with plasma ATX activity, but it is still unclear whether there is a contribution of its product LPA to pruritus, and which other pruritogens may play are role^10^. Our present study shows that cholestatic mice do not scratch more than healthy mice. In fact, in both cholestatic *Atp8b1* mutant mice and ANIT-treated WT mice, scratch activity decreases rather than the expected increase, despite an increase in plasma ATX activity (Fig. 1). This lack of scratch activity has been consistently overlooked and/or neglected in rodent studies addressing pruritogenic signaling pathways in cholestasis^14,32–37^. In contrast, Cipriani et al.^7^ also could not find increased scratch behavior in cholestatic (ANIT-treated) mice.

In addition, experimental enhancement of plasma ATX activity in the circulation had no effect on scratch behavior. Together, these results indicate that in mice, cholestasis does not induce pruritus (at least not in *Atp8b1* mutant mice and ANIT-treated mice) and enhanced plasma ATX activity does not translate into increased scratch activity under the chosen experimental conditions.

So far, very few research groups measured itch in rodents in an automated and long term assay. A review of the literature learns that mice and rats are usually taken out of their cages and placed individually in an empty transparent cage. In such studies, scratch activity was measured at daytime using video recording followed by counting by investigators, usually for a very limited time period (e.g. 60 min). Measuring scratch activity in an automated assay removes all human influences, as well as bias and errors. An additional important advantage of our method is that the mice permanently stay in the same cage, together with a littermate (without magnets). By measuring for 12 h during the night, with multiple consecutive nights per experimental condition, we were able to include all activity during the waking period of the mice. We strongly advocate this automated, unbiased, undisturbed and long-term assay for this behavioral type of study. The *Atp8b1* mutant mouse is an excellent model for the human inherited disease, progressive familial intrahepatic cholestasis type 1 (PFIC1), which is (in humans), characterized by frequent and strong pruritus. Scratch activity of the *Atp8b1* mutant mouse has not been reported before. Our present study shows that cholestasis in *Atp8b1* mutant mice led to a decreased rather than increased scratch activity. We saw similar results in WT mice treated with the cholestasis-inducing toxicant ANIT. Hence, our present data indicate that, with regard to cholestatic itch, mouse studies cannot be simply translated to human (patho-) physiology.

### Intrahepatic cholestasis

Another method to induce cholestasis is bile duct ligation (BDL)^38,39^, which has a similar time-span and duration as cholestasis in *Atp8b1* mutant mice on a 0.1% CA diet. However, BDL is a harsh extrahepatic method for cholestasis, leading to liver fibrosis, bile duct proliferation and finally cirrhosis and ascites^40^. Some studies report that BDL gives spontaneous scratching in mice^41,42^, but not in rats^43^. Noteworthy, all of these scratch measurements where performed during daytime for 60 min or less.

### ANIT

Treatment with the hepatotoxicant alpha-naphthylisothiocyanate (ANIT) is an established model to induce intrahepatic cholestasis in rodents; it leads to acute cholestasis with severe damage of bile duct epithelial cells and consequent obstructive cholestasis. Studies with regard to itch induced by ANIT treatment are conflicting. Recently, Meixiong et al. reported significant scratch behavior in mice (129svj/C57BL6J mixed background) that received ANIT for five days (25 mg/kg/day p.o.)^12^. Conversely, Cipriani et al. treated mice (C57BL/6NCrl background) with ANIT (25 mg/kg/day) for 10 days but did not observe significant scratch behavior compared to WT or TGR5^-/-^ mice that underwent the same treatment^7^. In our hands, treatment of WT mice with 50 mg/kg ANIT compared to treatment with vehicle for five days led to a significant increase in plasma ATX activity, but a decrease in both scratch activity and total movements (Fig. 2; suppl. Figure 4B). A study of Zhou et al. shows that ANIT-treatment affects serotoninergic concentrations in serum and prefrontal cortex, and that ANIT-treated mice display anxiety-like behavior^44^, which we did not measure. This might be an explanation though for our findings of reduced scratch activity and total movements. Further research is necessary to elucidate whether serotoninergic levels and anxiety affect scratch activity in all cholestatic mouse models.

### TGR5

Studies from the Bunnett group showed that mice with a transgene for the bile salt receptor TGR5 have increased spontaneous short-term scratch activity, and that intradermal injection of the bile salt deoxycholic acid (DCA) caused TGR5-dependent scratch activity in mice^36,37^. In these experiments, unconjugated DCA was chosen as a ligand at concentrations (± 6 mmol/L) much higher than those needed for TGR5 activation (EC_50_ ± 100 µmol/L). Particularly unconjugated DCA, which is a strong and cell-permeable detergent, will lead to massive calcium influx in cells, if not to local cell death by dissolution of cell membranes. In contrast and similar to our findings, Cipriani et al. previously showed a decreased acute scratch response upon intradermal injections of DCA in cholestatic mice^7^. Together with our observation that cholestatic mice do not display more long-term scratch behavior than control mice, this challenges the proposal that TGR5-mediated signaling plays an important role in cholestatic itch. The conditions applied (bile salt feeding to (pregnant) *Atp8b1* mutant mice) lead to very substantial elevations of bile salts (i.e. TGR5 agonists) in the circulation (Table 1). Yet, these animals do not display increased scratching (Fig. 1). Conditions of less elevated plasma bile salt levels have been reported to cause TGR5 signaling in mice (e.g.^45,46^). It therefore remains to be proven whether TGR5 signaling plays a causative role in long-term cholestatic itch in vivo and in patients. An alternative explanation for decreased scratch activity with increased bile salt levels during cholestasis in mice might be that the TGR5 pathway is desensitized during chronic cholestasis, as suggested by Cipriani et al.^7^. Our observation of reduced spontaneous scratch behavior in cholestatic mice (Figs. 1, 2) would fit with this hypothesis and illustrates the difficulty of translating human data to mice and vice versa. Additionally, mice have increased bile salt hydroxylation, which reduces bile salt toxicity and leads to a different bile salt pool^2^. Possibly, mice also have different downstream pathways of itch sensors, compared to humans.

### MRGPRX4

Recent studies have postulated a role for the human MRGPRX4 receptor in cholestasis-associated itch signaling^12,47^. The secondary bile salt DCA induced a strong activation of the MRGPRX4 receptor, while primary bile salts like TCDCA and GCDCA, which are much more prominent in plasma of cholestatic patients, showed lower affinity to MRGPRX4^47^. Scratch activity was found to be increased in a mouse model expressing the human MRGPRX4 receptor after injection of bile salts^12^. Similar to the in vitro studies, the secondary bile salt DCA showed the strongest effect on scratch activity. However, DCA is present in plasma only in very low concentrations (the majority of bile salts in plasma is conjugated with glycine or taurine). Furthermore, in clinical cases of isolated hypercholanemia itch is not occurring. Thus, in patients with a deficiency of the hepatic bile salt uptake transporter, NTCP, plasma bile salt concentrations can be as high as 100–1000 µM without the patients complaining of itch^48–51^. Furthermore, in many patients (particularly in patients with primary biliary cholangitis, PBC) itch is the first symptom of the disease when plasma bile salts are hardly increased. Conversely, pruritus often subsides in the later, more progressed, phase of PBC when serum bile salts are strongly increased^52^. Hence, bile salts activating the MRGPRX4 receptor may contribute to itch sensation, but are certainly not the dominant factor and their role in cholestasis-associated itch needs to be further assessed.

### Estrogens

This study shows that normal intake of dietary phytoestrogens do not induce scratch activity in *Atp8b1* mutant mice (no CA diet) (Fig. 3). During pregnancy, plasma estrogen and progesterone levels increase^53^, and might contribute to cholestasis^54^. Estrogen and progesterone may be metabolized into cholestasis-inducing compounds and undergo enterohepatic circulation, possibly leading to pruritus^55^. Sulphated progesterone metabolites, which are increased in the serum of women with ICP, correlate with itch intensity^14^. One of those sulphated progesterone metabolites has been shown to activate TGR5 and lead to pruritus when injected intradermally in mice^14^. Compounds with a bile salt-like structure, including steroids like progesterone metabolites, are able to inhibit plasma ATX activity (Langedijk et al. manuscript in preparation), which may explain the observed decrease in ATX activity upon phytoestrogen feeding (Fig. 3).

### ATX activity

In principle, our observation of a lack of change in scratch behavior in cholestatic mice fits very well with our observation that ATX is much less elevated in cholestatic and pregnant mice compared to cholestatic and pregnant humans (Fig. 4). In mice, endogenous plasma ATX activity was elevated no more than 2.1-fold under the experimental conditions tested in Fig. 1C,D. However, in patients with cholestasis and women with ICP, the increases in plasma ATX activity are much higher (Fig. 4) (16.8-fold in women with ICP)^9,10,14,24^.

In order to test whether the lower ATX levels in plasma of mice are the cause of the absence of itch, we induced a fivefold increase in plasma ATX activity in mice by transduction with AAV8-ATX constructs (Figs. 5B, 7B). This neither induced scratch activity (Figs. 5C, 7C), nor did the observed scratch activity decrease during subsequent treatment with an ATX inhibitor (Fig. 7D,E). Simultaneous induction of cholestasis with 0.1% CA in *Atp8b1* mutant mice transduced with AAV8-CMV-ATX further induced plasma ATX activity but still, long-term scratch activity decreased (Fig. 5B,C) compared to non-cholestatic animals. The conclusion from these experiments is that high plasma ATX activity per se is insufficient to evoke scratch behavior in mice. From the observation that women with uncomplicated pregnancy (without pruritus) have significantly higher plasma ATX activity than cholestatic patients with itch (Fig. 4), we propose that increased LPA signaling at most potentiates itch sensation, rather than causing it.

Overall, we provided evidence that intrahepatic cholestasis in mice (in *Atp8b1* mutant mice caused by CA feeding and in WT mice caused by ANIT-treatment), does not lead to itch and cannot serve as a model of cholestatic pruritus in human patients in general and PFIC type 1 patients in particular. In mice, an increase in systemic ATX activity is not sufficient to cause chronic pruritus.

The fact that cholestatic mice show less, rather than more spontaneous scratch activity, complicates further research on the mechanism of cholestatic itch in mice. A next step should be taken in developing humanized mouse models, to accomplish a more human bile salt pool or higher levels of circulating pruritogens that are to be found in the bile and plasma of human patients with itch.

## Supplementary Information

Supplementary Information

## Abbreviations

AAV8: Adeno-associated virus serotype 8
ALP: Alkaline phosphatase
ALT: Alanine aminotransferase
ANIT: Alpha naphthyl isothiocyanate
AST: Aspartate aminotransferase
Atp8b1: ATPase phospholipid transporting 8b1
ATX: Autotaxin
BDL: Bile duct ligation
CA: Cholic acid
CMV: Cytomegalovirus
DCA: Deoxycholic acid
HLP: Hybrid liver-specific promoter
ICP: Intrahepatic cholestasis of pregnancy
IHC: Immunohistochemistry
PFIC: Progressive familial intrahepatic cholestasis
TGR5: Transmembrane G-protein coupled receptor 5
