# Supplementary material for: Reduced spontaneous itch in mouse models of cholestasis
Source: Sci Rep. 2021 Mar 17;11:6127. doi: 10.1038/s41598-021-85660-1 (PMC7969945; doi:10.1038/s41598-021-85660-1)
Supplement: Supplementary file 1 — Supplementary Information [file 41598_2021_85660_MOESM1_ESM.pdf]

Supplementary figures

Reduced spontaneous itch in mouse models of cholestasis

Authors:

Jacqueline Langedijk[1§], Ruth Bolier[1§], Dagmar Tolenaars[1], Lysbeth ten Bloemendaal[1], Suzanne Duijst[1], Dirk de Waart[1], Ulrich Beuers[1], Piter Bosma[1], Ronald Oude Elferink[1*]

Affiliations:

[1]Amsterdam UMC, University of Amsterdam, Tytgat Institute for Liver and Intestinal Research, Amsterdam Gastroenterology Endocrinology Metabolism Research Institute, Amsterdam, The Netherlands

[§]These authors contributed equally to this work

[*]Ronald Oude Elferink (ORCID: https://orcid.org/0000-0002-1779-0833)

Correspondence author address:

Tytgat Institute for Liver and Intestinal Research

Meibergdreef 69-71

1105 BK Amsterdam, The Netherlands

Tel. +31-20-5663828

E-mail: r.p.oude-elferink@amsterdamumc.nl

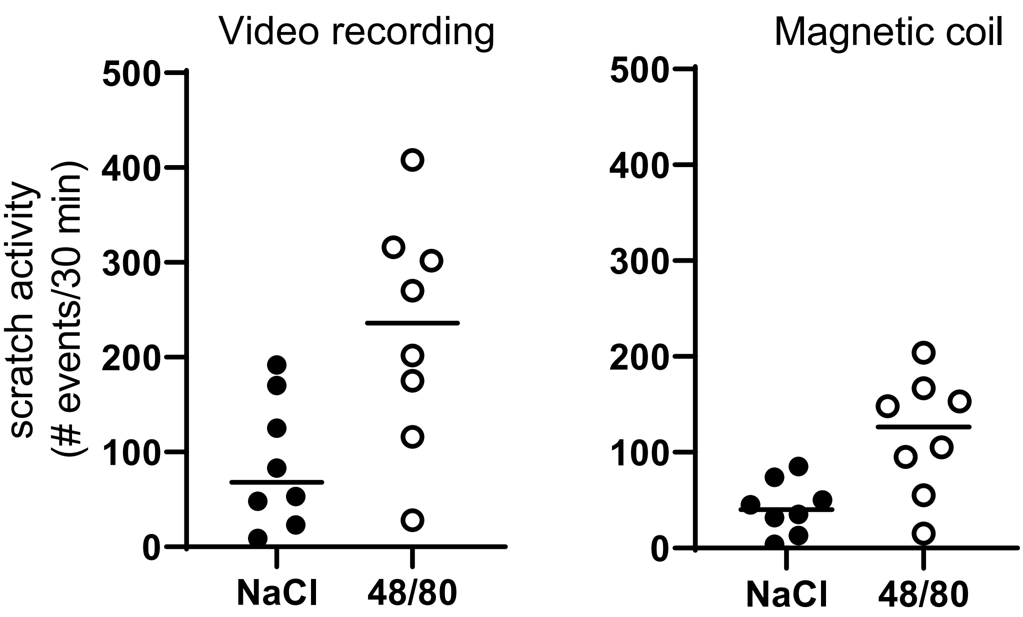

Supplementary figure 1A: Scratch activity (in seconds) measured during 30 minutes immediately after intradermal injection of 0.9% NaCl (50µl) or C48/80 (7µg in 50µl) in WT mice (n=7) or *Atp8b1* mutant mice (n=6) that where on a 0.1% CA diet for 17 days. Bars depict mean ± SD. Statistics: paired t-test; * p-value <0.05, ** p-value <0.01.

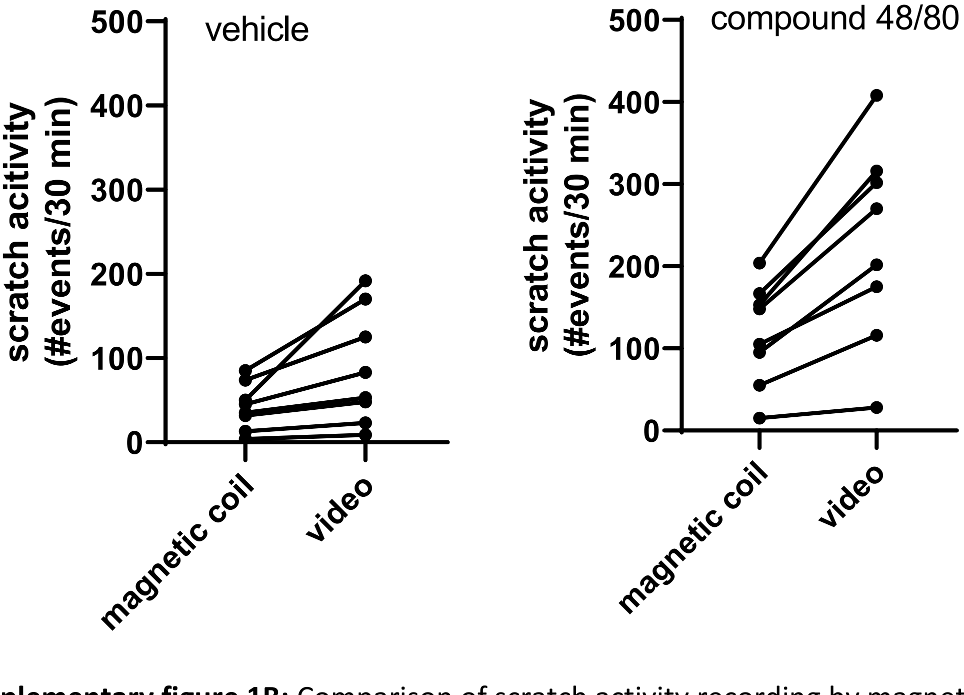

Supplementary figure 1B: Comparison of scratch activity recording by magnetic coil or videorecording. Scratch activity (in events) was measured during 30 minutes immediately after intradermal injection of vehicle (saline) or C48/80 (7µg) in WT mice (n=8) and scratch events were monitored simultaneously by magnetic oil and by video recording

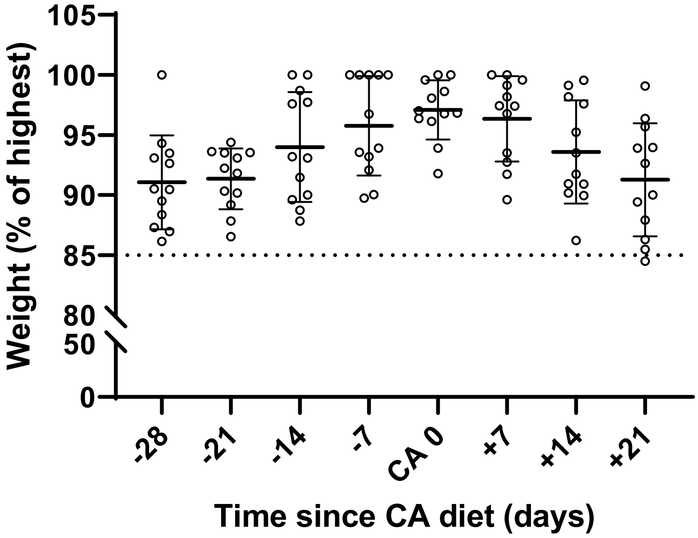

Supplementary figure 2: Weight (in percentage of highest measured weight of each animal) of *Atp8b1* mutant mice (n=12) before and after 0.1% CA diet (8-10 weeks old at start of measurements). Time in days relative to the start of CA diet. The dotted line indicates the critical humane end point of 15% weight loss. Bars depict mean ± SD.

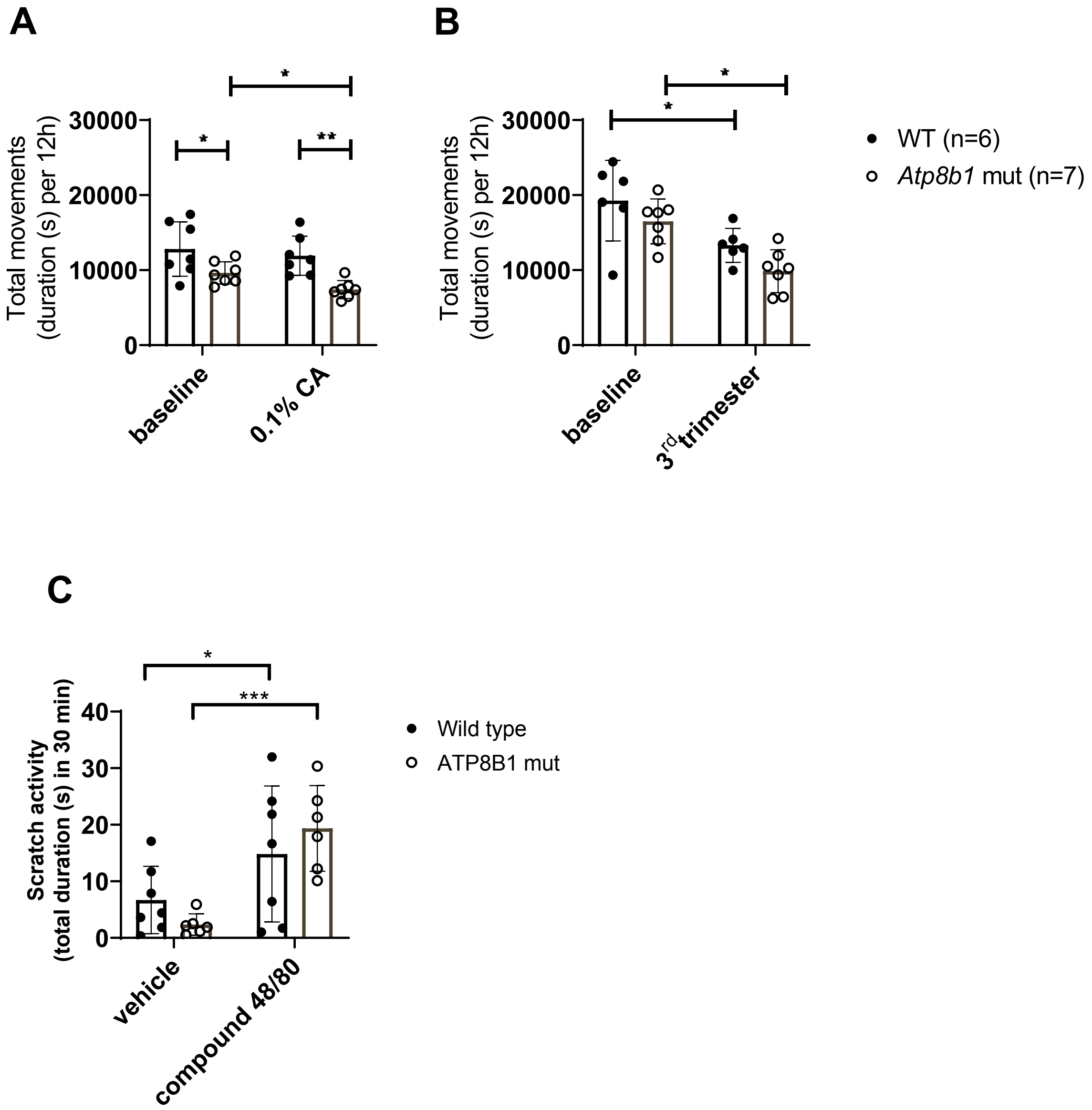

Supplementary figure 3: Total movements (as mean duration in seconds over four consecutive nights per experimental condition) in wild type mice on a semi-synthetic reference diet (n=7 females; n=6 pregnant females) and *Atp8b1* mutant mice on a semi-synthetic reference diet with supplementation of 0.1% CA from day 12 of pregnancy (n=7 females; n=7 pregnant females), before (**A**) and during pregnancy (**B**).

Wild type and cholestatic Atp8b1 mice are equally sensitive to the pruritogen compound 48/80 (**C**). Mice were fed a 0.1% CA-supplemented diet as indicated in Fig. 1. Subsequently, the animals received an intradermal injection of compound 48/80 (7μg in 50 μl) and scratch activity was measured for 30 min. Bars depict mean ± SD. Statistics: two-way ANOVA followed by Sidak's post hoc test; within genotypes all symbols are shown, between genotypes only symbols are shown when significant; ns: not significant, * p-value <0.05, ** p-value <0.01.

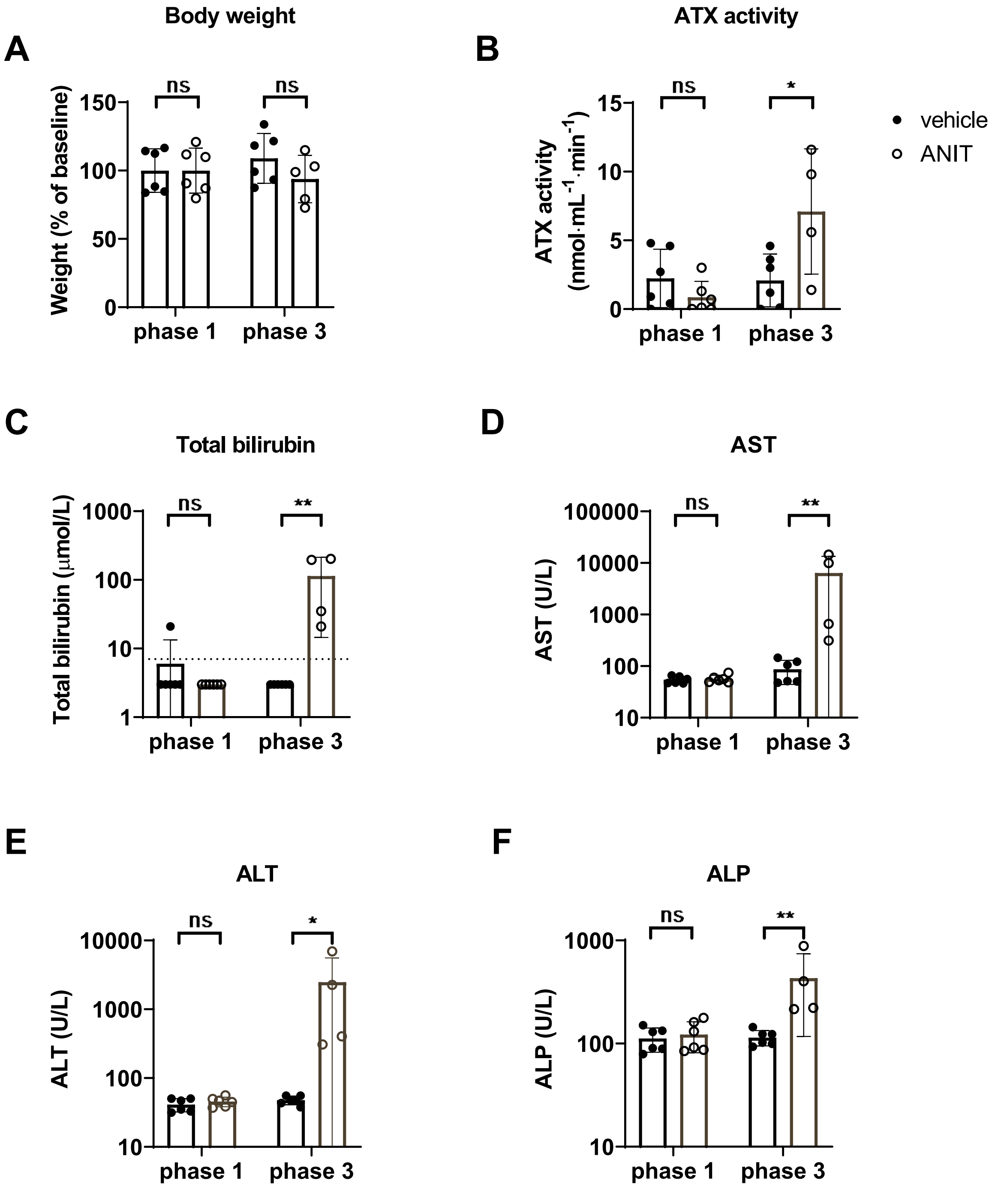

Supplementary figure 4: (**A**) Body weight (in percentage of baseline), (**B**) plasma ATX activity (in nmol·mL^{-1}·min^{-1}), (**C**) plasma total bilirubin (µmol/L), (**D**) plasma aspartate aminotransferase (AST) (U/L), (**E**) plasma alanine aminotransferase (ALT) (U/L), (**F**) plasma alkaline phosphatase (ALP) (U/L). All measured in WT mice during baseline scratch measurement (phase 1) and after oral treatment of vehicle (n=8) or ANIT (n=8) (phase 3). Bars depict mean ± SD. Statistics: two-way ANOVA followed by Sidak's post hoc test; ns not significant, * p-value <0.05, ** p-value <0.01.
